# Immune Gene *INHBA* is Associated With Osteoarthritic Cartilage Damage and May Mediate the Temporal Activation of the TGF‐β/p38 MAPK Pathway: Integrating Multiomics Machine Learning and Experimental Validation

**DOI:** 10.1155/mi/8787726

**Published:** 2026-06-30

**Authors:** Jiahao Sun, Qi Sun, Yiqiang Chen, Xin Li, Xu Cui, Yongkui Zhang, Wenpeng Xie

**Affiliations:** ^1^ Shandong University of Traditional Chinese Medicine, Jinan, 250014, Shandong Province, China, sdutcm.edu.cn; ^2^ Taian First People’s Hospital, Taian, 271000, Shandong Province, China; ^3^ Affiliated Hospital of Shandong University of Traditional Chinese Medicine, Jinan, 250014, Shandong Province, China, sdutcm.edu.cn

**Keywords:** experimental validation, immune genes, machine learning, molecular docking, multiomics analysis, osteoarthritic cartilage

## Abstract

**Objective:**

This study aimed to screen and identify osteoarthritis (OA)‐related core immune genes, elucidate the mechanism underlying the temporal activation of key signaling pathways mediated by these genes, and screen potential therapeutic compounds, thereby providing a theoretical basis for the precision diagnosis and targeted intervention of OA.

**Methods:**

Four sets of OA cartilage transcriptomic datasets with normal controls (NCs) were downloaded from the Gene Expression Omnibus (GEO) database. After batch correction, a training set and two independent validation sets were constructed to screen immune‐related differentially expressed genes (Immune‐DEGs). A total of 113 combined models were built based on 12 basic machine learning algorithms; the models were optimized via 10‐fold cross‐validation and evaluated in the validation sets to identify important immune genes. Subsequent functional enrichment analysis was performed to clarify the enriched signaling pathways. Core immune genes were identified by integrating Shapley additive explanations (SHAP) analysis and weighted gene coexpression network analysis (WGCNA), and single‐cell pseudotime analysis was used to decipher their dynamic expression patterns during OA progression. Finally, real‐time quantitative PCR (RT‐qPCR) and western blot (WB) were employed to verify the expression changes of core genes and key pathway proteins in cell models and OA animal cartilage tissues with different lesion severities. Additionally, drug repurposing was conducted using the DSigDB database to screen potential therapeutic compounds, and molecular docking and molecular dynamics simulations were performed to validate the binding ability of clinically commonly used drugs to core targets.

**Results:**

A total of 12 immune‐DEGs significantly associated with OA were screened. Through systematic evaluation of multiple models, an exploratory OA classification model based on 10 important immune genes was constructed, showing good classification ability in the limited samples of this study. Functional enrichment analysis revealed that these important immune genes were significantly enriched in the TGF‐β signaling pathway. Integration of SHAP and WGCNA analyses identified *INHBA* as a key hub gene associated with OA, and single‐cell pseudotime analysis indicated that *INHBA* expression was sustainably upregulated with OA progression. In vitro and in vivo experimental validation showed that *INHBA* mRNA levels in human chondrocytes and *INHBA* mRNA levels in rat cartilage tissues continuously increased with the aggravation of OA lesions; WB results demonstrated that total TGF‐β1 protein exhibited a temporal change of first increasing and then decreasing, while *INHBA* and downstream phosphorylated p38 MAPK protein levels continuously rose during disease progression, suggesting a temporal activation switch between the TGF‐β pathway and the p38 MAPK pathway. Drug repurposing yielded 10 potential therapeutic compounds including progesterone; molecular docking indicated that diclofenac, celecoxib, glucosamine, and chondroitin sulfate could stably bind to core targets, and molecular dynamics simulations further confirmed the stable binding conformation between celecoxib and INHBA protein.

**Conclusion:**

This study successfully constructed an exploratory OA classification model based on 10 important immune genes, demonstrating that the core immune gene *INHBA* is strongly associated with OA progression and may contribute to cartilage degeneration in OA by mediating the temporal activation of the TGF‐β/p38 MAPK pathway.

## 1. Introduction

Osteoarthritis (OA) is a chronic joint disease characterized by articular cartilage degeneration, subchondral bone sclerosis, and synovial inflammation [1, 2]. It not only causes joint pain, stiffness, and functional impairment in patients but also imposes a heavy health and economic burden on society. Traditionally regarded as a condition caused by mechanical wear and tear, recent studies have demonstrated that chronic low‐grade inflammation mediated by the immune system plays a pivotal role in its initiation and progression [3]. The local joint microenvironment in OA exhibits complex immune dysregulation, including abnormal infiltration and activation of various immune cells, as well as the upregulated expression of inflammatory factors, chemokines, and matrix‐degrading enzymes. These changes collectively accelerate the degradation of the cartilaginous extracellular matrix (ECM), disrupt the cartilage‐bone metabolic balance, and promote the generation of pain signals [4]. Therefore, in‐depth exploration of the regulatory mechanisms underlying the OA immune microenvironment and identification of key immune molecular markers and targets are crucial for understanding the pathological essence of OA and developing novel intervention strategies.

Despite the growing recognition of the role of immune inflammation in OA, significant challenges remain in its clinical translation. Currently, the diagnosis of OA primarily relies on clinical manifestations and imaging examinations, which can only confirm the diagnosis when structural damage is already evident. The lack of early biological markers with high sensitivity and specificity limits the early warning and dynamic monitoring of the disease [5]. With the rapid advancement of high‐throughput sequencing technology, integrating bulk transcriptomic and single‐cell resolution data enables us to more systematically identify differentially expressed genes (DEGs) from heterogeneous cell populations and uncover key genes and signaling pathways involved in OA progression through functional enrichment analysis [6]. However, research on systematically constructing OA prediction models and screening therapeutic targets based on immune‐related genes is relatively underdeveloped. There is an urgent need to combine multidimensional omics data with advanced machine learning algorithms to advance the development of precision diagnosis and targeted therapy strategies for OA.

To address this gap, the present study integrates multiple sets of OA cartilage transcriptomic datasets and single‐cell sequencing data from the Gene Expression Omnibus (GEO) database. We first screened immune‐related DEGs (Immune‐DEGs) and systematically applied various machine learning algorithms to construct an exploratory OA classification model, aiming to identify immune genes most relevant to OA cartilage damage. Furthermore, functional enrichment analysis was performed to reveal the regulated signaling pathways, and pseudotime analysis was used to decipher the evolutionary patterns of core immune genes during disease progression. Real‐time quantitative PCR (RT‐qPCR) and western blot (WB) were employed to verify the dynamic expression differences of genes and proteins in osteoarthritic cartilage of varying severity. On this basis, potential therapeutic compounds were screened through drug repurposing databases, and molecular docking and molecular dynamics simulations were utilized to evaluate the interaction between clinically commonly used drugs and targets. Collectively, these efforts provide a new theoretical basis and strategic support for the mechanism elucidation, early diagnosis, and targeted therapy of OA.

## 2. Methods

### 2.1. Data Acquisition and Preprocessing

OA‐related gene expression data were downloaded from the GEO database. Detailed information on all datasets is summarized in Supporting Information 1: Table S1. The training set was constructed by merging two human articular cartilage transcriptomic datasets: GSE169077 and GSE178557. All samples were collected from articular cartilage tissues, and patients had no history of other joint diseases or systemic inflammatory disorders. The two OA datasets were subjected to background correction, quantile normalization, and expression value calculation using Bioconductor R packages and then merged to form the training set. The ComBat‐seq algorithm was applied to remove batch effects between the two datasets, and the effectiveness of batch correction was verified by principal component (PC) analysis (PCA). DEGs in the training set were identified using the limma R package, with the screening criteria set as Benjamini–Hochberg (BH) adjusted *p*  < 0.05 and a 1.5‐fold change in expression (logFC = 0.585). In addition, the GSE235610 and GSE246425 datasets were processed separately using the ComBat algorithm to eliminate technical biases between different batches, resulting in two independent validation sets.

### 2.2. Construction of Machine Learning Models

All machine learning model construction, hyperparameter tuning, and performance evaluation were implemented using the R programing language (version 4.3.1), with core R packages including glmnet, caret, xgboost, randomForest, and e1071 for algorithm implementation and result analysis.

First, the immune‐related gene set was obtained from the ImmPort database (version 2024‐01, https://www.immport.org/), which contains 2483 manually curated genes involved in immune system processes, immune response regulation, cytokine signaling, and inflammatory pathways. To completely eliminate data leakage and ensure the independence of model evaluation, we strictly implemented the entire feature selection and modeling workflow within a nested cross‐validation (NCV) framework, with the full pipeline detailed as follows:

Step 1: NCV framework setup

We adopted a 10‐fold outer loop for model performance evaluation and a 5‐fold inner loop for feature selection and hyperparameter tuning, with no data overlap between the training and test partitions in any fold. All feature preprocessing steps, including differential expression analysis and immune‐DEG filtering, were performed exclusively within the training partition of each outer cross‐validation fold, with no access to test partition data or outcome labels at any point.

Step 2: Feature selection within each outer fold

In the training partition of each outer fold, DEGs between OA and normal samples were identified using the limma R package, with screening criteria set as BH adjusted *p*  < 0.05 and |logFC| ≥ 0.585. Immune‐related DEGs (Immune‐DEGs) were defined as the intersection of DEGs and the ImmPort immune gene set, which were used as input features for subsequent modeling in the fold.

Step 3: Hyperparameter tuning and model training

A total of 113 algorithm combination strategies were tested based on 12 basic machine learning algorithms (including ridge regression, lasso regression, support vector machine, logistic regression, random forest, and gradient boosting machine) combined with Elastic Net regularization and XGBoost. The area under the receiver operating characteristic (ROC) curve (AUC) was used as the primary optimization metric. Key hyperparameters were optimized via grid search in the inner 5‐fold cross‐validation: the alpha parameter of elastic net regularization (0.1–1, step = 0.1), the tree depth (3–10, step = 1) and learning rate (0.01–0.2, step = 0.01) of the gradient boosting machine. The hyperparameter combination with the highest mean AUC across inner folds was selected for each outer fold.

Step 4: Model performance evaluation

The optimal model from the inner loop was applied to the held‐out test partition of the corresponding outer fold to calculate the performance metrics. After completing all 10 outer folds, the overall model performance was summarized, and the optimal feature set and hyperparameters with the highest mean AUC across all outer folds were identified.

Step 5: Final model construction and external validation

The final linear discriminant analysis (LDA) model was retrained on the entire training set using the optimal hyperparameters and the 10 key immune genes were identified via the NCV framework. The classification ability of the model was evaluated on the training set using confusion matrices, ROC curves, and AUC values, while the generalization performance was verified on two completely independent external validation sets (GSE235610 and GSE246425), which were never used in the training or cross‐validation processes. It should be noted that the perfect AUC of 1.0 in GSE246425 is mainly attributed to the small sample size and low heterogeneity of this dataset rather than the true generalizability of the model.

All statistical tests for model performance were two‐sided, and exact *p*‐values were reported where applicable. On this basis, Gene Ontology (GO) and Kyoto Encyclopedia of Genes and Genomes (KEGG) enrichment analyses were conducted on the 10 key immune genes, with BH correction applied to adjust for multiple testing (adjusted *p*  < 0.05 was considered statistically significant).

### 2.3. Screening of Core Immune Genes

#### 2.3.1. Model Interpretability Analysis

Shapley additive explanations (SHAPs) were used to analyze and interpret the results of the machine learning models [7]. SHAP is a tool for explaining model predictions, which can more intuitively demonstrate the contribution of each gene to the model results.

#### 2.3.2. Construction of Weighted Gene Coexpression Network Analysis (WGCNA)

The WGCNA package in R was used to construct an OA cartilage coexpression network based on the merged expression matrix of GSE235610 and GSE246425, which included a total of 32 independent human articular cartilage samples (18 OA samples and 14 normal control [NC] samples). Previous studies have confirmed that WGCNA has sufficient statistical power to identify stable gene modules with a sample size of ≥20; thus, our sample size is adequate for this analysis.

First, the ComBat algorithm was applied to eliminate batch effects between the two datasets prior to WGCNA construction, and the effectiveness of batch correction was verified via PCA. The optimal soft threshold *β* = 12 was determined via scale‐free network analysis (scale independence = 0.9), and the adjacency matrix and topological overlap matrix (TOM) were constructed through *β*‐power transformation. Gene modules were divided using the dynamic hybrid cutting method (minimum module size = 100 genes), and module eigengenes (ME) were calculated. The correlation between ME and the clinical traits (OA vs. normal) was analyzed using Pearson correlation coefficients, and the MEdarkred module, which showed the strongest positive correlation with OA (*r* = 0.72, *p*  < 0.001), was defined as the hub module.

To evaluate the robustness of the identified modules, we performed module preservation analysis using the training set (GSE169077 + GSE178557) as the reference dataset. The results showed that the MEdarkred hub module had a high preservation *Z*‐score (Zsummary = 12.3, *p*  < 0.001), indicating excellent stability and reproducibility across independent datasets. Regarding the use of validation datasets for WGCNA: the two datasets were only used for model performance validation in the machine learning section and were used as an independent discovery cohort for WGCNA to avoid overfitting to the training set, which does not compromise the independence of the results.

#### 2.3.3. Core Immune Genes

The core immune genes were obtained by taking the intersection of the results from machine learning and WGCNA, combined with SHAP analysis results.

### 2.4. Experimental Validation

#### 2.4.1. Single‐Cell Sequencing Data Validation

The staged single‐cell transcriptomic dataset GSE104782 of clinical OA was downloaded from the GEO database for core validation. In this study, we uniformly used the following OA stage abbreviations throughout the entire manuscript, with definitions based on the internationally recognized Kellgren–Lawrence (K–L) grading system (the gold standard for clinical OA severity assessment) and widely accepted rat OA surgical modeling time points in the field:

POA: Early‐stage OA, defined as K–L grade 1–2 for human clinical cartilage samples and 4 weeks after surgical modeling for rat OA model samples.

MOA: Middle‐stage OA, defined as K–L grade 2–3 for human clinical cartilage samples and 8 weeks after surgical modeling for rat OA model samples.

EOA: Late‐stage OA, defined as K–L grade 3–4 for human clinical cartilage samples and 12 weeks after surgical modeling for rat OA model samples.

This dataset was originally generated from 10 independent human articular cartilage donor samples, including three POA, three MOA, and four EOA patient samples, which were staged by its authors based on the K–L grading system mentioned above.

Raw data were subjected to stringent and unified quality control and filtering using R packages (Seurat and dplyr) to remove low‐abundance genes and low‐quality cells. The unified quality control criteria were set as follows: genes expressed in at least three cells, 200–10,000 genes detected per cell, and mitochondrial gene percentage < 10%. After strict quality control and filtering, a total of 1578 high‐quality valid chondrocytes were retained for subsequent analysis, including 623 cells from three POA patients, 318 cells from three MOA patients, and 637 cells from four EOA patients.

PCA was performed on the normalized expression matrix. The optimal number of PCs was jointly determined via JackStraw plot and Elbow Plot analysis (Supporting Information 2: Figure S1A, B): the JackStraw plot showed that the first 20 PCs were all statistically significant with extremely low *p*‐values (*p*  < 0.001), indicating that these PCs stably captured the true biological variation in the dataset; the Elbow Plot further revealed that the standard deviation of PCs reached a distinct plateau after the 20th PC, suggesting that subsequent PCs only reflected random noise without biological significance. Furthermore, the cumulative variance explained by the first 20 PCs exceeded 80% of the total gene expression variation, which was sufficient to retain the core biological information. Therefore, the first 20 PCs were selected for tSNE and UMAP dimensionality reduction and clustering analysis with a resolution of 0.6.

Cell clusters were annotated based on the expression characteristics of classic chondrocyte marker genes, and DEGs between OA stages and between cell clusters were calculated using the Wilcoxon rank‐sum test (Seurat standard workflow), with Bonferroni correction for multiple testing and a filtering threshold of genes detected in at least 10% of cells in the tested group to minimize false‐positive results. This analytical framework is the gold standard for OA single‐cell transcriptomic research and has been widely adopted in top‐tier studies in the field, including the original publication of this dataset [8]. The pseudotime analysis pipeline of Monocle3 [9] was employed to decipher the temporal expression dynamics of the core immune gene *INHBA* during OA progression, and the VECTOR tool was used for unsupervised trajectory inference [10]. Additionally, simulated gene knockout of the core target was performed using scTenifoldKnk to investigate its regulatory impacts on downstream gene networks.

#### 2.4.2. Sample Preparation

Cell experiment: To simulate the pathological states of OA with varying severity, interleukin‐1β (IL‐1β) was used to intervene in in vitro cultured chondrocytes to construct an in vitro OA inflammatory injury model [11]. The chondrocyte line used in this study was the C28/I2 human chondrocyte line, a widely recognized and commonly used chondrocyte model in OA in vitro research due to its stable chondrocyte‐specific phenotype and high consistency with primary chondrocyte biological characteristics. To avoid phenotypic drift caused by excessive cell passage and eliminate potential interference of passage times on experimental results, only third‐generation (P3) chondrocytes in the logarithmic growth phase were selected for all experiments. Well‐grown P3 chondrocytes were randomly divided into a blank control group (without IL‐1β intervention) and different concentration IL‐1β treatment groups (3 ng/mL, 6 ng/mL, and 10 ng/mL), with three technical replicates per group and three independent repeated experiments. The concentration gradient was selected based on previous widely accepted OA in vitro studies: 3 ng/mL IL‐1β induces mild inflammatory response and matrix metabolic imbalance in chondrocytes, corresponding to the POA phenotype; 6 ng/mL IL‐1β triggers significant upregulation of catabolic genes (MMP13) and downregulation of anabolic genes (COL2A1), corresponding to the MOA phenotype; and 10 ng/mL IL‐1β causes severe chondrocyte inflammatory injury, hypertrophic differentiation, and ECM degradation, corresponding to the EOA phenotype [12]. The cells were incubated in a 37°C, 5% CO_2_ incubator for 24 h. After intervention, the culture supernatant was discarded, and the cells were gently rinsed twice with precooled PBS. Cells were harvested by trypsin digestion, and total RNA and total protein were extracted separately to simulate the chondrocyte phenotypes of OA with different damage degrees.

Animal experiments: All animal experiments were approved by the Animal Ethics Committee of the Affiliated Hospital of Shandong University of Traditional Chinese Medicine (Approval Number: SDSZYYAWE20240408001) and strictly performed in accordance with the ARRIVE 2.0 guidelines and the Guidelines for the Ethical Review of Laboratory Animal Welfare. Experimental rats were divided into the blank control group (NC), POA, OA MOA, and EOA, with six rats per group to ensure statistical power. The OA groups were established using a combined surgical approach of anterior cruciate ligament transection (ACLT) + medial meniscectomy (DMM) + medial collateral ligament transection [13], while the blank control group only underwent skin incision to expose the joint cavity followed by direct suture without ligament or meniscus manipulation. Enrofloxacin (5 mg/kg/day) was injected subcutaneously for three consecutive days after surgery to prevent infection, butorphanol (0.1 mg/kg) was administered for analgesia 2–3 days postoperatively. Rats were encouraged to move freely to avoid joint stiffness. Rats in the POA, MOA, and EOA groups were sacrificed at 4, 8, and 12 weeks after surgery, respectively; the blank control group was sacrificed simultaneously with the EOA group to eliminate time interference. After sacrifice, the bilateral tibial plateau articular cartilage and surrounding joint tissues were completely isolated. Micro‐CT was used to detect subchondral bone remodeling and osteophyte formation; hematoxylin and eosin (HE) staining was performed to observe the morphological structure of joint tissues; safranin O (SO)–fast green staining was used to evaluate the degree of cartilage proteoglycan loss; and OARSI scoring was combined to verify the modeling effect and reliability of OA staging. Meanwhile, articular cartilage tissues were collected, ground in liquid nitrogen, and part of the tissue was used for total protein extraction (quantified by the BCA assay) and total RNA extraction (for RT‐qPCR detection of INHBA mRNA expression).

#### 2.4.3. RT‐qPCR

RT‐qPCR was used to detect the mRNA expression levels of *MMP13*, *COL2A1*, and *INHBA* genes in in vitro chondrocytes with different damage degrees and in vivo rat articular cartilage at different OA stages. *GAPDH* was used as the reference gene, and the 2^−ΔΔCt^ method was employed to calculate the relative expression levels of target genes so as to verify the effectiveness of OA model construction and analyze the temporal expression patterns of target genes.

##### 2.4.3.1. RT‐qPCR Detection of Cell Samples

C28/I2 chondrocytes in each group after 24‐h IL‐1β intervention were collected, and total cellular RNA was extracted using the TRIzol reagent, with strict avoidance of RNase contamination throughout the process. The concentration and purity of RNA were determined using a NanoDrop nucleic acid detector to ensure that the A260/A280 ratio was between 1.8 and 2.0. A total of 1 μg of qualified total RNA was used for the reverse transcription reaction with the PrimeScript RT Master Mix kit to synthesize cDNA, which was stored at −20°C for later use. Using cDNA as the template, amplification reactions were performed on the QuantStudio 3 real‐time fluorescent quantitative PCR system with the SYBR Premix Ex Taq reagent. The total reaction volume was 20 μL, including 10 μL of SYBR Green mix, 0.4 μL of each forward and reverse primer, 2 μL of the cDNA template, and 7.2 μL of ddH_2_O. The reaction conditions were set as follows: predenaturation at 95°C for 30 s, followed by 40 cycles of amplification at 95°C for 5 s and 60°C for 30 s. After cycling, melting curve analysis was performed to verify the specificity of primer amplification. Each group had three technical replicates, and the experiment was independently repeated three times. The average value was used for subsequent statistical analysis. The primer sequences used are as follows: *MMP13* (Forward: CTTGCTGGCTGCTGCTGCTGCC; Reverse: CACCGCCATGAATGGAGTGCTGCTG; 108 bp); *COL2A1* (Forward: TGGACGATCAGCGGCAAACA; Reverse: GCGGTGAGGTCTTGGTGGTTCT; 149 bp); *INHBA* (Forward: GGCTACCATGACAACCTGTC; Reverse: CACAGTAAGGCGTTCCATCA; 150 bp); and *GAPDH* (Forward: GGAGCGAGATCCCTCCAAAAT; Reverse: GGCTGTTGTCATACTTCTCATGG; 197 bp).

##### 2.4.3.2. RT‐qPCR Detection of Animal Samples

Articular cartilage tissues from the tibial plateau of rats in each group were ground thoroughly into powder in liquid nitrogen. Total tissue RNA was extracted using the TRIzol reagent. Subsequent RNA purity and concentration detection, cDNA reverse transcription, qPCR amplification system, and conditions were consistent with those for cell samples. GAPDH was used as the reference gene, and the 2^−ΔΔCt^ method was employed to calculate the relative expression levels of each target gene. Each group included six rat samples, with three technical replicates per sample. The average value was used for statistical analysis.

#### 2.4.4. WB

WB was used to detect the expression levels of TGF‐β1, p38 MAPK, p‐p38 MAPK, and INHBA proteins in in vitro chondrocytes and articular cartilage of rats with different OA stages in vivo. *GAPDH* served as the internal reference protein to analyze the temporal activation pattern of pathway proteins.

##### 2.4.4.1. WB Detection of Cell Samples

Adherent chondrocytes in each group were lysed with RIPA lysis buffer containing PMSF and phosphatase inhibitors (volume ratio of 98:1). After sufficient lysis, the supernatant was collected, and the protein concentration was determined using the BCA assay. The BCA working solution was prepared at a ratio of *A*:*B* = 50:1. PBS and 5× protein loading buffer were used to adjust the protein sample concentration to 1–2 mg/mL, followed by denaturation in boiling water for 10 min. A total of 20 μg of total protein was separated by 12% SDS‐PAGE gel electrophoresis. After electrophoresis, the NC membrane was equilibrated in precooled transfer buffer for 30 min and electrotransferred at a constant current of 400 mA at 4°C for 1 h. After transfer, the membrane was blocked with TBST solution containing 5% nonfat milk at room temperature for 2 h and then washed three times with TBST (5 min each time). Subsequently, the membrane was incubated with the corresponding primary antibodies at 4°C overnight. All primary antibodies were diluted in TBST containing 2.5% BSA, including TGF‐β1 (1:1000), p38 MAPK (1:1000), p‐p38 MAPK (1:2000), and INHBA (1:1000). After thorough washing with TBST, the membrane was incubated with HRP‐conjugated goat anti‐rabbit IgG secondary antibody (1:10,000) at room temperature for 1 h. Following another round of thorough TBST washing, the membrane was reacted with the HRP chemiluminescent substrate for 2 min, and signals were collected by short exposure on a Tanon 5200 automatic chemiluminescence imaging system. Gray value quantitative analysis was performed using ImageJ software.

##### 2.4.4.2. WB Detection of Animal Samples

Powdered articular cartilage tissues of rats (ground in liquid nitrogen) in each group were mixed with RIPA lysis buffer containing PMSF and phosphatase inhibitors (tissue:lysis buffer volume ratio of 1:4). After thorough homogenization and ultrasonic lysis, the supernatant was collected by centrifugation at 12,000 g for 10 min at 4°C. Subsequent steps, including protein concentration quantification, SDS‐PAGE electrophoresis, membrane transfer, blocking, primary and secondary antibody incubation, and chemiluminescent development, were consistent with those for cell samples. GAPDH was used as the internal reference protein. ImageJ software was employed to determine the gray value of target protein bands and calculate the relative expression levels of target proteins. Each group included six rat samples, and the experiment was independently repeated three times. The average value was used for statistical analysis.

#### 2.4.5. Proteomics

To further verify the expression characteristics of the core immune gene *INHBA* at the protein level, an independent group of animals was raised in parallel for proteomic detection in this study. The experiment included an operation group (OA model group, *n* = 3) and a sham operation group (NC group, *n* = 3). The modeling method (the operation group received a combined surgical approach of ACLT + DMM + medial collateral ligament transection; the sham operation group only underwent skin incision to expose the joint cavity, followed by direct suture) and postoperative care measures (subcutaneous injection of enrofloxacin at 5 mg/kg/day for three consecutive days to prevent infection, administration of butorphanol at 0.1 mg/kg for analgesia 2–3 days postoperatively, and encouragement of free movement to avoid joint stiffness) were identical to those in the animal experiment described in Section 2.4.2. The independent group design was used to eliminate sample interference between multiple detection items, and the sample size of *n* = 3 met the requirements of proteomics for sample depth and sensitivity in differential protein identification. Rats in both groups were sacrificed synchronously at 8 weeks after surgery. Articular cartilage tissues from the tibial plateau were collected completely, quickly frozen in liquid nitrogen, and stored at −80°C for later use.

The proteomic detection process was as follows: cartilage tissues were ground into powder in liquid nitrogen, and lysis buffer containing 1% Triton X‐100 and protease inhibitors was added at a ratio of 1:4 (v/v). After ultrasonic lysis, the supernatant was collected by centrifugation at 12,000 g for 10 min at 4°C, and the protein was quantified by the BCA assay. Equal amounts of protein were precipitated with acetone and reconstituted in 200 mM TEAB buffer. Trypsin digestion was performed at 37°C overnight at a trypsin:protein ratio of 1:50 (m/m). After reduction with 5 mM DTT (56°C, 30 min) and alkylation with 11 mM IAA (room temperature, protected from light, 15 min), peptides were separated by gradient elution using a Vanquish neo ultra‐high‐performance liquid chromatography system (mobile phase A: 0.1% formic acid + 2% acetonitrile; mobile phase B: 0.1% formic acid + 90% acetonitrile) at a flow rate of 700 nL/min. Detection was performed using an Orbitrap Exploris 480 mass spectrometer in the DIA mode, with an NSI ion source voltage of 2300 V, a FAIMS compensation voltage of −45 V, a primary scan resolution of 60,000, a secondary scan resolution of 15,000, and an HCD collision energy of 27%. Raw data were identified and subjected to label‐free quantification (LFQ) using MaxQuant software (referencing the UniProt rat database). Differential proteins were screened with a fold change > 1.3 or < 1/1.3 and *p*  < 0.05, with a focus on changes in the protein expression level of the core target INHBA.

### 2.5. Drug Screening, Molecular Docking, and Molecular Dynamics Simulation

Drug screening based on important immune genes from machine learning results: The DSigDB database (https://maayanlab.cloud/Enrichr/enrich) was used for drug screening based on intersecting genes to identify potential therapeutic drugs [14]. Clinically commonly used drugs were selected, and their active ingredient chemical structures were obtained from PubChem (https://pubchem.ncbi.nlm.nih.gov/). The three‐dimensional structures of important immune gene proteins were retrieved from the RCSB Protein Data Bank (PDB) (http://www.pdb.org/). AutoDock Tools (v1.1.2) was used for protein and molecular preprocessing, format conversion, and binding pocket analysis. After determining the docking sites, AutoDock Vina was employed for molecular docking, and coordinates were set for docking validation. Results were expressed as binding affinity values, and some interactions were visualized using PyMOL. A lower binding energy indicates a stronger interaction between the protein and the molecule.

Based on the receptor–ligand complexes obtained from molecular docking, further molecular dynamics simulation studies were conducted. After preprocessing and splitting the complexes using PyMOL, Sobtop was used to generate small‐molecule topology files (gro/itp) based on the AMBER force field, and GROMACS (2020.3‐MODIFIED) with the amber99sb‐ildn force field was used to construct the protein topology. After ion and solvation treatment of the system, energy minimization (steepest descent method, 5000 steps), NVT temperature rise equilibrium (100 ps, position restraint), and NPT pressure equilibrium (100 ps, 1 bar, 300 K) were performed sequentially. Finally, an unrestrained production simulation of 100 ns was executed, and dynamic properties of the system, such as root mean square deviation (RMSD), root mean square fluctuation (RMSF), radius of gyration (*R*_*g*_), Gibbs free‐energy landscape, and number of hydrogen bonds, were analyzed based on the trajectory.

### 2.6. Statistical Methods

GraphPad Prism 9.0 statistical software was used for all data statistical analysis. The Shapiro–Wilk test was first performed to verify the normal distribution of the data. For normally distributed data, measurement data were expressed as the mean ± standard deviation (*x* ± *s*). One‐way analysis of variance (one‐way ANOVA) followed by Tukey’s post hoc test for multiple comparisons correction was used for comparisons among multiple groups. For data that did not conform to a normal distribution, the nonparametric Kruskal–Wallis *H* test was used for intergroup comparisons. Exact *p*‐values were reported for all comparisons. All multiple testing was corrected using the BH method. An adjusted *p*‐value <0.05 was considered statistically significant, and adjusted *p*  < 0.01 was considered highly statistically significant.

## 3. Results

### 3.1. Identification of INHBA as the Core Target

#### 3.1.1. Analysis of OA Cartilage Transcriptomic Data

**Figure 1:**
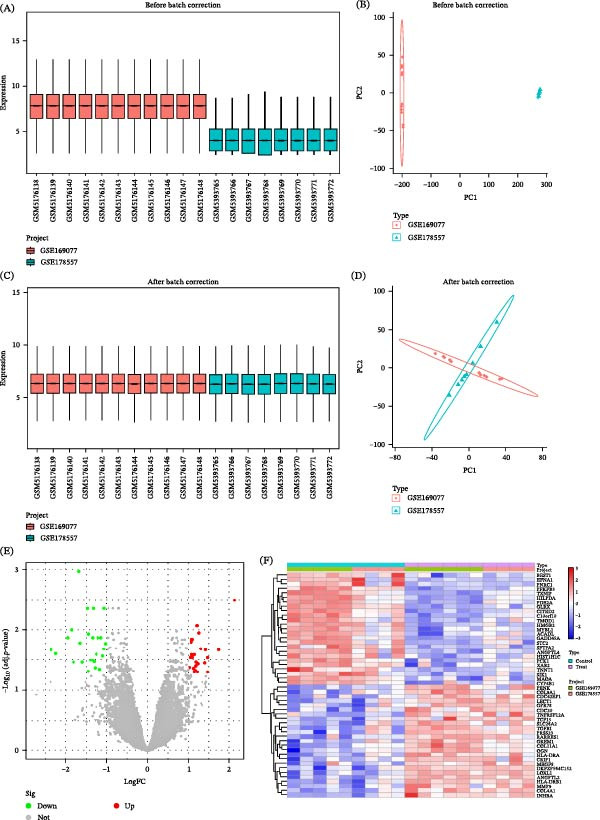
Boxplot and PCA plots before and after batch effect correction. (A, B) Boxplot and PCA plot of the distribution of gene expression values before batch effect correction. (C, D) Boxplot and PCA plot of the distribution of gene expression values after batch effect correction. (E) Volcano plot of differentially expressed genes between OA and normal samples. (F) Heat map of differential gene expression.

Transcriptomic data from two OA datasets (GSE169077 and GSE178557) were merged and subjected to batch correction (Figure 1A–D). After confirming that the expression value distribution tended to be consistent, differential expression analysis was performed. The results showed that a total of 50 genes exhibited significantly differential expression between OA patients and the control group, yielding OA‐related DEGs (DEGs‐OA) (Figure 1E,F).

#### 3.1.2. Construction of Machine Learning Models for Intersecting Targets and Enrichment Analysis

Joint analysis was performed between DEGs‐OA and the immune‐related gene set [15], and we successfully identified 12 OA‐associated Immune‐DEGs, which were used as the input feature set for subsequent machine learning model construction.

Based on these 12 Immune‐DEGs, we constructed 12 basic machine learning models and tested a total of 113 algorithm combination strategies. Model performance was optimized via 10‐fold cross‐validation and systematically evaluated in the training set and two independent external validation sets (Figure 2A–C). The results showed that the LDA was identified as the optimal algorithm for the OA prediction model, which was constructed based on 10 key immune genes (*OGN, MMP9*, *STC2*, *ANGPTL2*, *INHBA, HLA-DRB1*, *TNFRSF12A, GREM1*, *ANGPTL4*, and *PENK*). In the training set, the LDA model achieved an AUC of 0.989, showing good classification ability in the limited samples of this study for OA and normal samples. Meanwhile, the model exhibited consistent performance in the two independent validation sets, with an AUC of 0.889 in GSE235610 and 1.0 in GSE246425 (Figure 2B). The confusion matrices further confirmed the high classification accuracy of the model in the training set and both validation sets (Figure 2C).

Figure 2Machine learning model construction and functional characterization of core immune genes. (A) AUC heat map of 113 algorithm combinations in the training cohort. (B) ROC curves of the optimal LDA model in the training set and two independent external validation sets. (C) Confusion matrices of the LDA model in the training set, GSE235610 validation set, and GSE246425 validation set. (D) Bubble plots of GO and KEGG functional enrichment results for the 10 core immune genes. (E) Volcano plot of differential expression of the 10 core immune genes between OA and normal samples. (F) Box plots of the expression levels of the 10 core immune genes in OA and normal cartilage samples. (G) Single‐gene ROC curves of the 10 core immune genes for OA discrimination. (H) Correlation network between core immune genes and OA joint‐infiltrating immune cells (linkET analysis). (I) Coexpression network of the 10 core immune genes. (J) Protein–protein interaction (PPI) network of the 10 core immune genes. Adjusted  ^∗^ *p*  < 0.05, adjusted  ^∗∗^ *p*  < 0.01, and adjusted  ^∗∗∗^ *p*  < 0.001.

**Figure:**
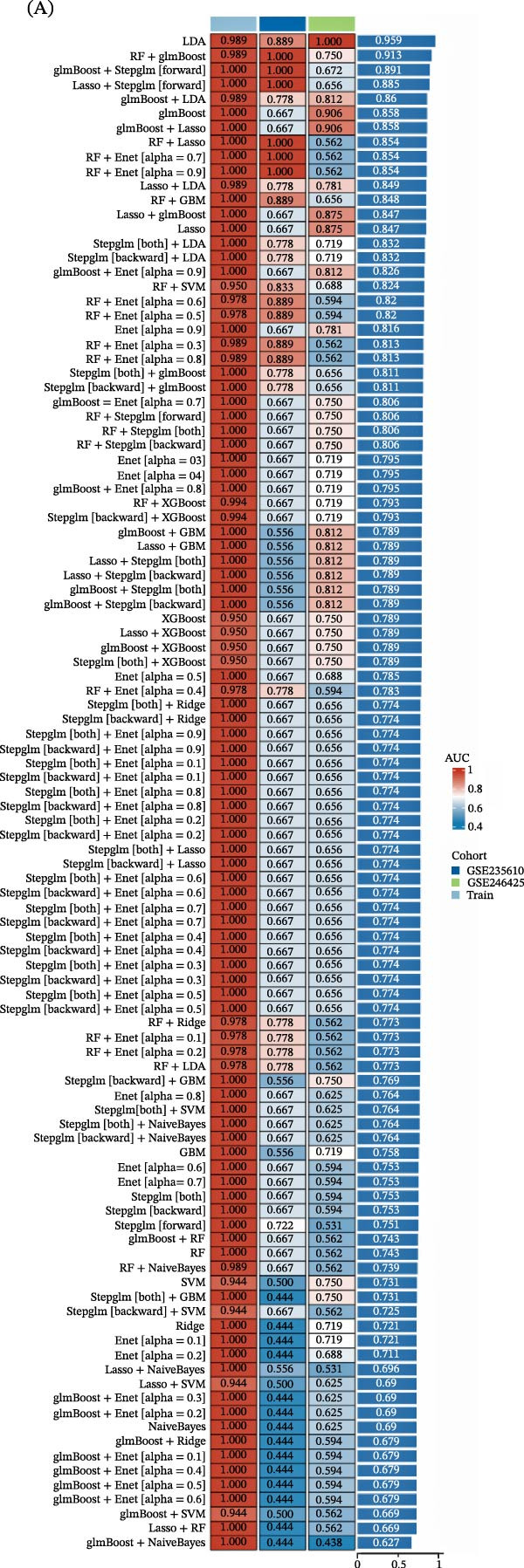

**Figure:**
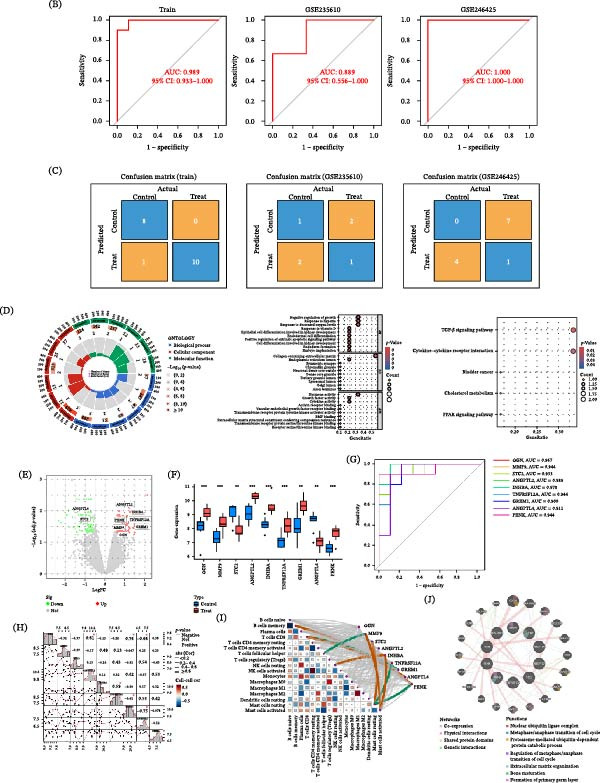

To clarify the biological functions of the 10 key immune genes, we performed GO and KEGG functional enrichment analyses (Figure 2D). KEGG pathway enrichment analysis revealed that these core genes were significantly enriched in critical pathways, including the TGF‐β signaling pathway, cytokine‐cytokine receptor interaction, and cholesterol metabolism. Among them, *INHBA* was identified as a core regulatory factor of the TGF‐β pathway, and *GREM1*, another key gene in the model, was also enriched in this pathway, both of which are involved in the regulation of cartilage catabolism and the inflammatory response. In addition, ANGPTL4 was found to participate in the homeostasis of the joint microenvironment by regulating lipid metabolism (cholesterol metabolism and the PPAR pathway). GO functional enrichment analysis further indicated that the 10 key immune genes were mainly involved in three core biological processes: (1) regulation of bone and cartilage development including GREM1 and OGN negatively regulating osteoblast differentiation and MMP9 driving collagen degradation and ECM remodeling; (2) inflammatory and immune responses, including INHBA‐mediated macrophage differentiation, TNFRSF12A‐involved apoptotic signaling pathways, and ANGPTL4‐regulated angiogenesis; and (3) stress response and metabolic regulation, such as STC2 and PENK responding to hypoxia and oxidative stress and ANGPTL4 negatively regulating lipase activity. Cellular component analysis highlighted that these genes were mainly localized in the collagenous ECM, while molecular function analysis showed significant enrichment in hormone/growth factor activity and cytokine receptor binding. In summary, the 10 key immune genes collectively drive the pathological process of OA by synergistically regulating bone metabolic imbalance, chronic inflammation, and lipid stress networks.

We further verified the expression characteristics and diagnostic efficacy of the 10 key immune genes. The volcano plot confirmed that all 10 core genes were significantly differentially expressed between OA and normal cartilage samples (Figure 2E), and the box plots further validated that the expression levels of these genes in OA cartilage tissues were significantly altered compared with the NC group (Figure 2F). ROC curve analysis showed that each core immune gene, when used as a single biomarker, exhibited excellent discriminative efficacy for OA (Figure 2G), among which *INHBA* showed outstanding diagnostic performance, providing a basis for its subsequent identification as the core hub gene.

Subsequently, we explored the coexpression pattern, immune cell correlation, and protein interaction network of the 10 key immune genes. Gene coexpression analysis revealed a tight regulatory network among the core genes (Figure 2I): the profibrotic factor GREM1 was strongly positively correlated with INHBA, MMP9 was significantly negatively correlated with the antiangiogenic factor ANGPTL4, and TNFRSF12A showed a positive synergistic effect with the antiapoptotic gene *PENK*, indicating that ECM degradation, fibrosis, and apoptotic pathways are closely linked during OA progression. The correlation network between core genes and immune cells showed that MMP9 was positively correlated with M1 macrophage infiltration, GREM1 was closely associated with activated CD4^+^ T cells, and ANGPTL4 significantly inhibited dendritic cell activity, suggesting that these core genes mediate chronic inflammation in OA by regulating macrophage polarization and T‐cell responses (Figure 2H). The protein–protein interaction (PPI) network constructed by GeneMANIA identified key interactions including HLA‐DRB1 with CDC27 (cell cycle regulation) and MMP9 with LAMB1 (basement membrane degradation), which were mainly enriched in biological modules such as ECM organization and bone maturation, further validating that the core genes drive OA progression by regulating matrix remodeling and osteogenic differentiation (Figure 2J).

Collectively, these results identified 10 key OA‐associated immune genes with robust classification and diagnostic efficacy and systematically clarified their biological functions and regulatory networks, laying a solid foundation for the subsequent screening and identification of the core hub driver gene of OA progression.

#### 3.1.3. Identification of the Core Immune Gene INHBA via Combined SHAP and WGCNA Analyses

**Figure 3:**
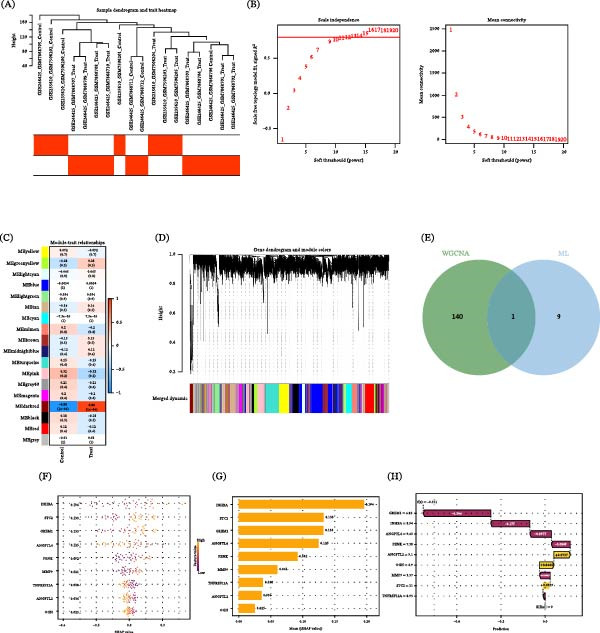
(A) Sample clustering, (B) optimal soft threshold, (C) relationship between main modules and OA, (D) cluster tree of differentially expressed genes, (E) Venn diagram, and (F–H) results of SHAP analysis.

WGCNA revealed that the optimal soft threshold (*β*) was 12 with a scale independence of 0.9. A hierarchical clustering tree of the network and coexpression modules was constructed, and all genes were divided into modules with each containing at least 100 genes. Among these modules, the MEdarkred coexpression module was significantly associated with OA (*p*  < 0.05) and contained 141 potential genes, indicating that hub genes of OA may exist in the MEdarkred coexpression module (Figure 3A–D).

Taking the intersection of the results from machine learning and WGCNA yielded the core target *INHBA* (Figure 3E). Furthermore, after model evaluation, all features and their importance from the built‐in feature importance analysis of the LDA machine learning model were identified (Figure 3F–H). Based on feature ranking, values, and frequency of occurrence in the model, *INHBA* was identified as a key hub gene associated with OA cartilage damage.

### 3.2. Validation

#### 3.2.1. Single‐Cell Sequencing Validation

Single‐cell transcriptomic datasets of cartilage tissues from the POA, MOA, and EOA stages of OA (GSE104782) were retrieved from the GEO database. Quality control and filtering of the data were performed using R packages such as Seurat and dplyr (Figure 4A). Cell annotation was completed based on the expression characteristics of classic marker genes, leading to the identification of seven major chondrocyte subpopulations: effector chondrocytes (ECs), homeostatic chondrocytes (HomCs), regulatory chondrocytes (RegCs), matrix‐producing chondrocytes (ProCs), prehypertrophic chondrocytes (preHTCs), fibrocartilage chondrocytes (FCs), and HTCs [8]. UMAP visualization clearly displayed the spatial aggregation features of each cell subpopulation (Figure 4B,C).

Figure 4Results of single‐cell sequencing differential analysis. (A) Quality control results. (B) Distribution of cell subsets in the two groups based on UMAP dimensionality reduction. (C) Distribution of cell types in UMAP space based on classical marker genes. (D) Trajectory analysis. (E–G) Pseudotemporal expression dynamics and pseudotemporal expression trajectory of the core immune gene *INHBA* in different chondrocyte subpopulations and different OA stages. (H) Unsupervised trajectory inference of chondrocytes along OA progression via the VECTOR tool. (I, J) Differences in *INHBA* expression across different OA stages (POA, MOA, and EOA).

**Figure:**
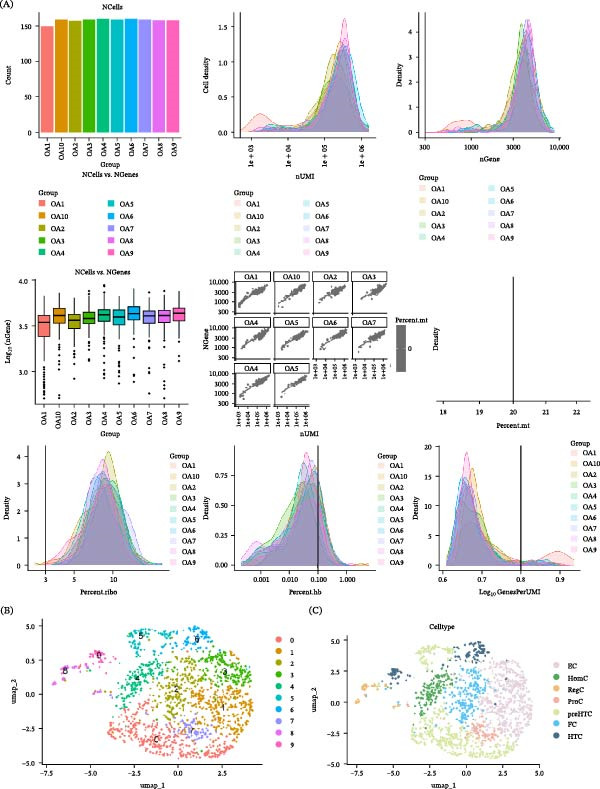

**Figure:**
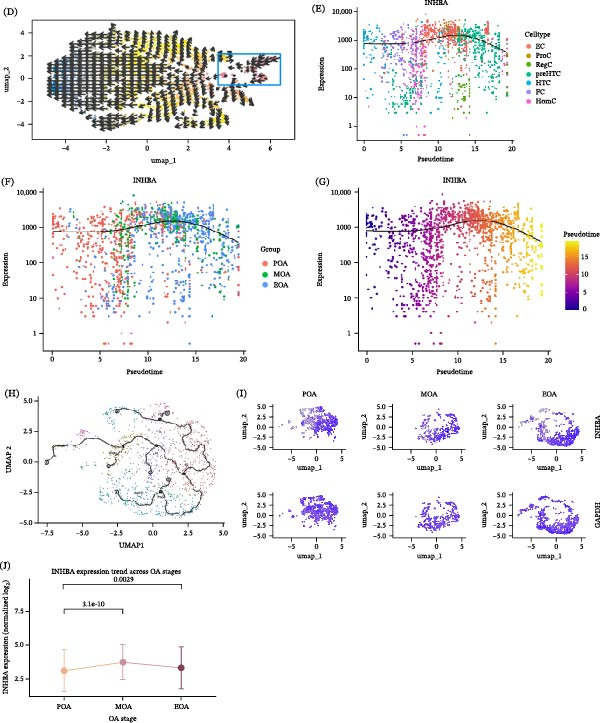

UMAP results of OA staging showed that cells from POA, MOA, and EOA samples exhibited a gradual clustering trend in the clustering space: POA‐stage cells were relatively concentrated; as the disease progressed to MOA and EOA stages, the cell distribution became increasingly dispersed, and the proportion of damage‐related subpopulations such as preHTC and FC significantly increased, indicating dynamic changes in the composition and phenotype of chondrocytes during OA progression (Figure 4H). The VECTOR tool was used for unsupervised trajectory inference, identifying HomC as the starting point of the developmental trajectory (Figure 4D). Pseudotime analysis results demonstrated that the expression level of INHBA was continuously upregulated with pseudotime progression; further validation via pseudotime expression analysis by cell subpopulation confirmed that INHBA expression was significantly higher in preHTC and FC than in stable subpopulations such as HomC. Pseudotime expression analysis by OA stage showed that INHBA expression in the EOA stage was significantly higher than that in the POA and MOA stages (Figure 4E–G).Quantification of *INHBA* expression differences across different OA stages revealed a gradual increase from POA to EOA (adjusted *p*  < 0.05). Notably, this upregulation trend was consistently observed in all individual patient samples within each OA stage, ruling out the possibility that the results were driven by a single outlier sample, confirming the robust temporal upregulation of *INHBA* expression with the progression of OA (Figure 4I,J).

**Figure 5:**
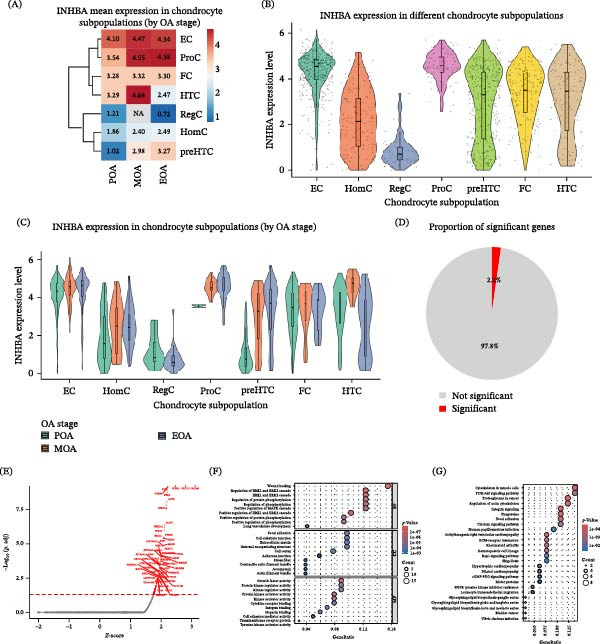
Expression characteristics of INHBA in chondrocyte subpopulations and functional validation after in silico knockout. (A) Heatmap of the mean expression of INHBA in chondrocyte subpopulations across different OA stages (POA/MOA/EOA). (B) Violin plot of INHBA expression in each chondrocyte subpopulation. (C) Stage‐stratified violin plot of INHBA expression in chondrocyte subpopulations by OA stage. (D) Pie chart of the proportion of significant genes after in silico INHBA knockout. (E) Volcano plot of differentially expressed genes after in silico INHBA knockout. (F) GO functional enrichment bubble plot of differentially expressed genes (BP, biological process; CC, cellular component; MF, molecular function). (G) KEGG pathway enrichment bubble plot of differentially expressed genes.

Combined with the subpopulation analysis of single‐cell sequencing data, we systematically characterized the expression profile of INHBA across chondrocyte subpopulations (Figure 5A–C). Consistent results from the heatmap (Figure 5A) and violin plot (Figure 5B) demonstrated that INHBA was persistently highly expressed in ECs, FCs, and preHTCs, while its expression was extremely low in HomCs and RegCs, indicating that the functional specificity of INHBA is concentrated in OA damage‐related cell subpopulations. The stage‐stratified violin plot (Figure 5C) further clarified that INHBA was already highly activated in EC and preHTC subpopulations at the POA, and with disease progression (from MOA to EOA), its high‐expression range gradually expanded to FC and HTCs subpopulations, driving the pathological activation throughout the entire course of OA. To further validate the core regulatory role of INHBA, we performed an in silico knockout analysis. The results showed that only 2.2% of genes exhibited significant expression changes after INHBA depletion (Figure 5D), suggesting a highly targeted regulatory effect of INHBA. The volcano plot (Figure 5E) revealed that following INHBA knockout, the core driver genes related to the MAPK signaling pathway, ECM degradation, and chondrocyte hypertrophy were all significantly downregulated. GO functional enrichment analysis (Figure 5F) further confirmed that OA pathological processes such as wound healing, ERK1/2 cascade, and positive regulation of the MAPK cascade were significantly inhibited after INHBA depletion, while KEGG pathway enrichment (Figure 5G) showed that the activation levels of key pathways involved in OA progression, including the PI3K–Akt signaling pathway, focal adhesion, and ECM–receptor interaction, were also markedly reduced.

Combined with the expression profile of INHBA in chondrocyte subpopulations and the results of in silico knockout analysis, these findings collectively demonstrate that INHBA functions as a key hub gene in OA progression. Specifically, INHBA is preferentially and persistently expressed in OA damage‐related chondrocyte subpopulations (EC, FC, and preHTC) throughout the entire course of the disease, and its depletion in silico leads to the significant inhibition of key pathological processes and signaling pathways closely associated with OA progression, such as MAPK/ERK pathway activation, ECM degradation, and inflammatory responses. These results collectively indicate that INHBA may contribute to OA cartilage degeneration by targeting and regulating the function of damage‐related chondrocyte subpopulations, thereby linking abnormal cell subpopulation activity to the dysregulation of core pathological signaling pathways.

#### 3.2.2. Experimental Validation

##### 3.2.2.1. Imaging and Histomorphological Validation of Animal Samples

Micro‐CT results showed that the knee joint bone structure of rats in the control group was intact, with smooth edges of the tibial plateau and femoral condyle and no osteophytes or abnormal subchondral bone remodeling. In the POA group, slight irregularities appeared on the articular bone surface, and subchondral bone density was slightly increased without obvious osteophyte formation. In the MOA group, newly formed osteophyte protrusions were observed at the edge of the tibial plateau, and subchondral bone sclerosis was aggravated. In the EOA group, the volume of osteophytes further increased, the joint space narrowed, and the subchondral bone showed significant abnormal remodeling, suggesting that subchondral bone lesions in OA progress in a disease‐dependent manner.

HE staining results revealed that the articular cartilage layer of the control group was clearly layered, with a uniformly arranged matrix and no fissures or defects. In the POA group, scattered tiny fissures appeared in the superficial layer of cartilage, with mild interruption of matrix continuity and no significant change in the cartilage layer thickness. In the MOA group, the cartilage fissures extended to the transitional layer, the matrix arrangement was disordered, and the cartilage layer thickness was reduced compared with that of the control group. In the EOA group, large‐area defects were observed in the cartilage layer, with the calcified layer directly exposed in some regions and severe destruction of the matrix structure, which was consistent with the pathological characteristics of cartilage damage in advanced OA.

SO–fast green staining and OARSI scoring results showed that the cartilage matrix of the control group was uniformly and deeply stained with SO. In the POA group, SO staining in the superficial layer of cartilage was weakened. In the MOA group, SO staining in the middle and deep layers of cartilage was significantly reduced, and vacuolar changes appeared in the matrix. In the EOA group, SO staining of cartilage almost disappeared, with only a small number of positively stained areas remaining. The differences in OARSI scores among all groups were statistically significant (all *p*  < 0.05), confirming the successful establishment of the OA staging model.

**Figure 6:**
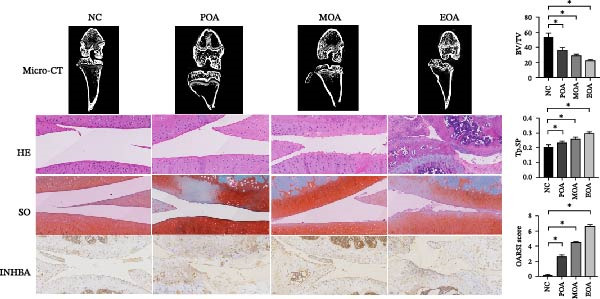
Pathological validation of the rat OA staging model and INHBA protein expression pattern in articular cartilage. Representative micro‐CT images, hematoxylin and eosin (HE) staining images, safranin O‐fast green (SO) staining images, and INHBA immunohistochemistry (IHC) images of rat knee joints in the normal control (NC) group, early‐stage OA (POA) group, middle‐stage OA (MOA) group, and late‐stage OA (EOA) group are shown. All images are from independent rat biological replicates (*n* = 6 per group). Corresponding quantitative analysis of bone volume/total volume (BV/TV), trabecular spacing (Tb.Sp), and OARSI histological scores are shown on the right. Scale bar = 1 mm for micro‐CT images, 50 μm for histological staining and IHC images. Exact *p*‐values for all comparisons are provided in the source data. Multiple testing was corrected using the Benjamini–Hochberg (BH) method. Adjusted  ^∗^ *p*  < 0.05.

Immunohistochemistry (IHC) results for INHBA showed almost no brownish‐yellow positive staining signal in the articular cartilage tissue of the control group. In the POA group, a small number of scattered weak positive staining was observed in the superficial chondrocytes. In the MOA group, the number of positively stained cells increased, the signal intensity enhanced, and the distribution range expanded to the transitional layer. In the EOA group, strong positive staining was observed in the residual cartilage cells and surrounding tissues, with widespread distribution of positive signals, suggesting that the expression of the INHBA protein is gradually enriched in the lesioned cartilage tissue with the progression of OA (Figure 6).

##### 3.2.2.2. Molecular‐Level Validation

To verify the successful construction of the IL‐1β‐induced chondrocyte inflammation model, we first detected the mRNA expression levels of classic OA phenotypic markers (*MMP13* and *COL2A1*). Compared with the blank control group, IL‐1β treatment concentration‐dependently induced the expression of the catabolic gene *MMP13* and concentration‐dependently inhibited the expression of the anabolic gene *COL2A1*. These results confirmed the successful establishment of the in vitro OA chondrocyte inflammation model, and different IL‐1β concentrations (3/6/10 ng/mL) could simulate the pathological phenotypes of POA, MOA, and EOA stages in vivo (Figure 7A,B).

Figure 7(A–C) RT‐qPCR results of cell models. (D) RT‐qPCR results from animal models. (E, F) WB results of cell and animal models. (G) Proteomics results. Exact *p*‐values for all comparisons are provided in the source data. Multiple testing was corrected using the Benjamini–Hochberg (BH) method. Adjusted  ^∗^ *p*  < 0.05, adjusted  ^∗∗^ *p*  < 0.01, and adjusted  ^∗∗∗^ *p*  < 0.001.

**Figure:**
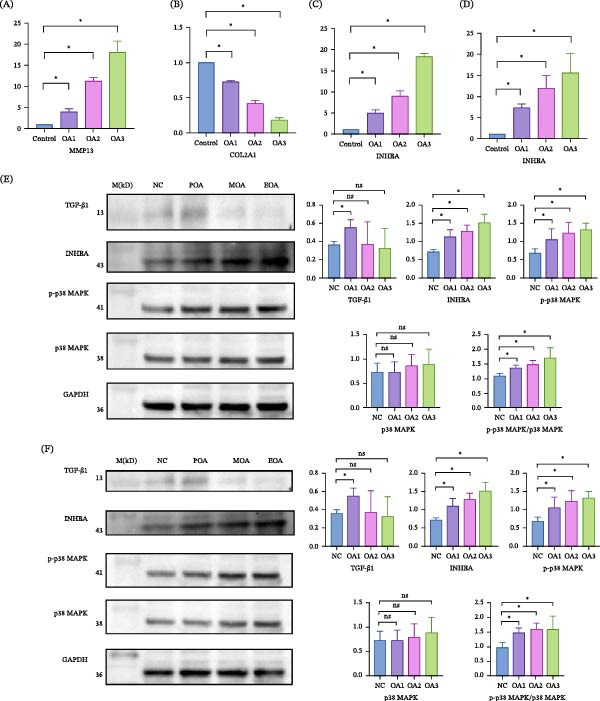

**Figure:**
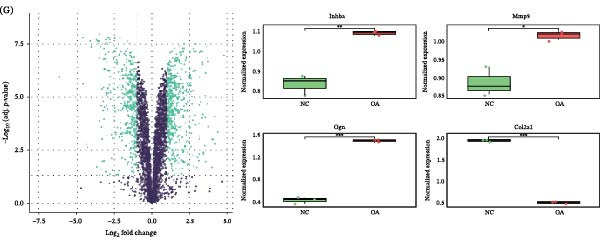

On the basis of model validation, we further detected the mRNA expression level of *INHBA* in the cell samples. Compared with the blank control group, the expression of *INHBA* in the 3, 6, and 10 ng/mL IL‐1β treatment groups gradually increased (*p*  < 0.05), and its expression trend was highly consistent with the dynamic changes of OA stages in vivo, suggesting the temporal expression characteristics of *INHBA* during OA progression (Figure 7C). RT‐qPCR results of animal samples further verified the temporal expression pattern of *INHBA* (Figure 7D): compared with the blank control group (NC), the mRNA expression level of *INHBA* was gradually upregulated with the progression of OA (*p*  < 0.05), which was completely consistent with the continuous upregulation trend of *INHBA* in single‐cell pseudotime analysis.

WB results showed that the protein expression of INHBA in cell samples was consistent with the mRNA level trend: with the increase of IL‐1β concentration, the protein expression of INHBA gradually increased (*p*  < 0.05); meanwhile, TGF‐β1 protein was significantly upregulated in the 3 ng/mL treatment group (*p*  < 0.05) and decreased to the control group level in the 10 ng/mL treatment group. Total p38 MAPK protein was stably expressed among all groups, but its phosphorylation level (p‐p38 MAPK) was continuously upregulated with the increase of IL‐1β concentration (*p*  < 0.001) (Figure 7E). In animal samples, the protein expression of INHBA was gradually upregulated with the progression of OA (*p*  < 0.05), which was highly consistent with the mRNA expression level. Detection of pathway‐related proteins showed that: TGF‐β1 protein was significantly upregulated in the POA group (*p*  < 0.05), slightly decreased in the MOA group but still higher than that in the control group (*p*  < 0.05), and returned to a level with no statistically significant difference from the control group in the EOA group; total p38 MAPK protein had no significant difference in expression among all groups, while the phosphorylation level of p‐p38 MAPK continued to increase with OA progression (*p*  < 0.05), suggesting that the activation degree of the p38 MAPK pathway was positively correlated with OA severity (Figure 7F).

##### 3.2.2.3. Proteomic Validation

Quantitative proteomic analysis results of cartilage tissues from the rat OA model (Figure 7G) showed that Col2a1 (type II collagen), a core component of the cartilaginous ECM, was significantly downregulated in the OA group (*p*  < 0.05); meanwhile, the cartilage matrix‐related protein Ogn was significantly upregulated in the OA group (*p*  < 0.05), further confirming the molecular phenotypic disorder of OA cartilage. The standardized expression level of the core target INHBA in the cartilage of the OA group was significantly higher than that in the NC group, directly verifying the specific high‐expression characteristic of INHBA in OA cartilage. The above results confirmed at the protein level that the high expression of INHBA in OA cartilage can directly drive the degradation of the cartilage ECM and promote the pathological process of OA cartilage damage.

### 3.3. Drug Prediction, Molecular Docking, and Molecular Dynamics Simulation

**Figure 8:**
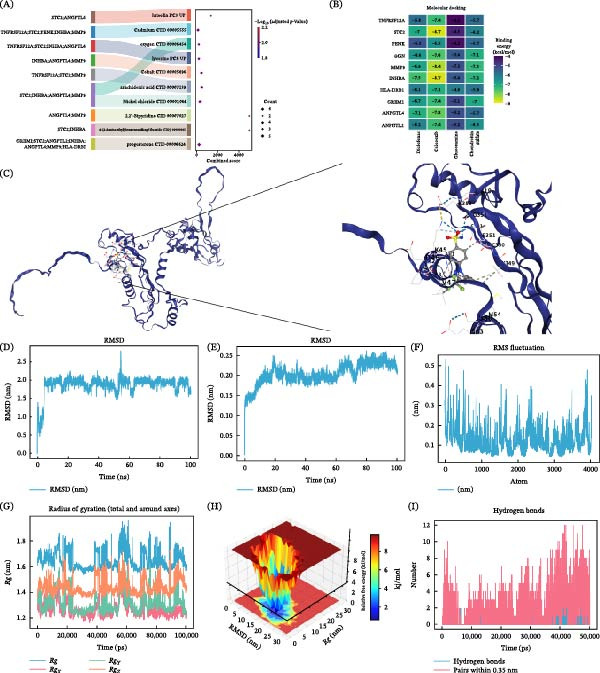
(A) Drug screening results. (B) Molecular docking results. (C) Celecoxib–INHBA complex. (D, E) RMSD analysis results of ligand–protein complex during simulation. (F) Protein RMSF analysis of ligand–protein complex during simulation. (G) Radius of gyration (*R*_*g*_) analysis of ligand–protein complex during simulation. (H) Free energy landscape of ligand–protein complex during simulation. (I) Results of hydrogen bond analysis of ligand–protein complexes during simulations.

Drug prediction analysis based on the key immune genes identified from machine learning results identified several potential therapeutic candidates for OA with significant statistical associations (Figure 8A). Progesterone showed the strongest comprehensive association, being significantly correlated with up to seven core genes, which suggests that it may have potential regulatory effects on the OA immune microenvironment. Additionally, 4‐(2‐aminoethyl)benzenesulfonyl fluoride and 2,2^′^‐bipyridine both exhibited extremely high specific association intensities. Furthermore, metal ions such as nickel chloride, cobalt, and cadmium also showed significant associations with multiple core genes. Plant active ingredients including lycorine and luteolin were also predicted as potential candidates. The essential fatty acid arachidonic acid was significantly correlated with TNFRSF12A, STC2, and MMP9. These prediction results provide preliminary clues for drug repurposing in OA, indicating that hormones (progesterone), specific enzyme inhibitors, metal ion modulators, reactive oxygen species (ROS)‐related interventions, plant alkaloids, and polyphenols may be worthy of further investigation as potential therapeutic agents targeting the core immune gene network of OA.

Diclofenac, celecoxib, glucosamine, and chondroitin sulfate were selected as molecular docking targets primarily based on their extensive clinical application in OA treatment. Diclofenac and celecoxib belong to nonsteroidal anti‐inflammatory drugs (NSAIDs), which alleviate joint inflammation and pain by inhibiting cyclooxygenase (COX) activity, serving as first‐line therapeutic drugs for OA. Glucosamine and chondroitin sulfate are slow‐acting drugs that improve cartilage structure, exerting protective effects by promoting cartilage matrix synthesis and inhibiting the activity of degrading enzymes. The selection of these four classic drugs for docking with core immune genes aims to verify their potential multitarget mechanisms of action and provide molecular‐level theoretical support for their known therapeutic effects.

Molecular docking results (Figure 8B) showed that the four drugs exhibited differential binding affinities (kcal/mol) with the 10 core immune genes. Celecoxib exhibited the strongest overall binding capacity, with binding energies below −6.5 kcal/mol for all targets, and the binding to INHBA was particularly significant. Diclofenac ranked second in binding strength, showing good binding to INHBA, ANGPTL4, and STC2. Glucosamine had relatively limited binding capacity, with moderate affinity only for OGN and INHBA. Chondroitin sulfate displayed strong interactions with structural genes.

To further verify the binding affinity between celecoxib and the target protein INHBA, as well as the structural stability of the complex after binding, 100 ns molecular dynamics simulations were performed on the celecoxib–INHBA complex (Figure 8C). Molecular dynamics simulation results demonstrated that the ligand–protein complex formed by celecoxib and INHBA had good structural stability: the RMSD of celecoxib remained stable throughout the simulation cycle (1–100 ns) with fluctuations below 1 nm. Analysis of the Rg further confirmed this conclusion, as the Rg values of each complex remained within a stable range during the 100 ns simulation without significant fluctuations, indicating that the binding of celecoxib to INHBA enabled the complex to achieve good internal structural and dynamic balance. RMSF analysis showed that the RMSF curves of the complexes were highly consistent with low overall structural flexibility; only the region of the first 2000 atoms at the N‐terminus of the protein exhibited significant fluctuation characteristics, indicating that this region is an intrinsic flexible domain of INHBA, and the binding of celecoxib altered the distribution of flexible regions and the overall flexibility of INHBA. In addition, the Gibbs free energy landscape derived from PCA showed that the celecoxib–INHBA complex had a clear minimum energy state. Hydrogen bond analysis results indicated that the ligand–INHBA complex stably maintained three hydrogen bonds throughout the simulation process (0–100 ns) (Figure 8D–I).

## 4. Discussion

This study employed a systematic research framework integrating multiomics screening, machine learning modeling, multidimensional validation, and drug prediction, focusing on the potential regulatory role of the immune gene *INHBA* in osteoarthritic cartilage damage via mediating the temporal activation of the TGF‐β/p38 MAPK pathway. By incorporating the consensus on OA pathological mechanisms and the latest research evidence in the field, we conducted in‐depth analyses from three dimensions: immune microenvironment regulation, temporal activation of core signaling pathways, and clinical translational value, thereby advancing the understanding of OA pathogenesis and providing novel directions for OA intervention.

INHBA serves as a key regulatory factor in the OA immune microenvironment and a key hub linking synovial inflammation to cartilage degeneration. Notably, recent studies have demonstrated that environmental pollutant exposure can drive chronic inflammatory tissue damage by abnormally regulating the expression of core immune genes, which provides an important cross‐validation for the pathogenic role of immune gene abnormalities in OA cartilage damage [16]. Through multiomics integration and machine learning screening, this study identified *INHBA* as a key hub gene associated with immune microenvironment dysregulation in OA cartilage, a conclusion highly consistent with the latest progress in OA pathological research [17]. Previous studies have indicated that OA is not merely a disease of mechanical wear‐induced cartilage degeneration but a systemic inflammatory disorder involving the dysregulated crosstalk between the synovium, cartilage, and subchondral bone [18], and recent research has further confirmed that INHBA plays a critical role in synovial–cartilage crosstalk during OA progression [19]. Repetitive overload exercise can induce high INHBA expression in the synovial lining layer, which then acts via a paracrine mechanism (secreted by the synovium and responded to by cartilage) to transiently promote chondrocyte anabolism, followed by sustained inhibition of matrix synthesis and activation of inflammatory pathways [20]. This aligns perfectly with the dynamic expression characteristics of INHBA observed in the present study—single‐cell sequencing revealed that INHBA is significantly enriched in OA damage‐related chondrocyte subpopulations and continuously upregulated with disease progression. Further subpopulation expression profiling clarified that INHBA was persistently and highly expressed in damage‐related chondrocyte subpopulations, including ECs, FCs, and preHTCs, while barely expressed in HomCs and RegCs that maintain cartilage homeostasis, clarifying the specific cellular targets of INHBA in OA progression. Meanwhile, in silico knockout analysis further validated its core regulatory function: INHBA depletion significantly downregulated the core driver genes related to MAPK pathway activation, ECM degradation, and chondrocyte hypertrophy and markedly inhibited the OA‐related pathological processes, which provided direct bioinformatics evidence for the prodamage role of INHBA in OA. This suggests that INHBA is not only an intrinsic signal for autonomous chondrocyte pathological activation but also an extrinsic inflammatory amplifier through synovial–cartilage crosstalk, which explains why high INHBA expression is highly synchronized with the vicious cycle of synovial inflammation and cartilage degradation in OA [21].

From the perspective of pathway regulatory networks, the TGF‐β signaling pathway, chemokine signaling pathway, and other pathways enriched by the 10 key immune genes do not function in isolation. Among these 10 genes, *INHBA* was consistently identified as the core hub gene via the triple cross‐validation of machine learning, WGCNA, and SHAP analysis; thus, being the focus of the subsequent mechanism exploration and functional validation of this study. This is consistent with the findings of a previous study showing that targeted inhibition of INHBA by rhapontigenin (RHAP) downregulates inflammatory factors (IL‐6 and IL‐8) and reduces the activity of matrix‐degrading enzymes (MMP1 and MMP13), further indicating that INHBA is a key cross‐regulatory target for immune inflammation and matrix degradation in OA [22–26].

*INHBA* mediates the temporal activation of the TGF‐β/p38 MAPK pathway, which acts as a functional switch from early compensatory protection to late‐stage pathological damage. The core innovation of this study lies in uncovering the dynamic mechanism by which *INHBA* drives the sequential activation of the TGF‐β/p38 MAPK pathway, a process that is highly matched with the progressive nature of OA cartilage damage and is fully supported by both our in vitro/in vivo experiments and existing literature evidence. However, it should be emphasized that the current data only demonstrate a strong correlation between *INHBA* expression and the temporal activation of the TGF‐β/p38 MAPK pathway. Definitive causal evidence requires additional gain‐ and loss‐of‐function studies, such as *INHBA* overexpression and knockdown in chondrocytes, as well as conditional knockout mouse models, to directly demonstrate that INHBA is necessary and sufficient to drive the sequential activation of this pathway and subsequent cartilage damage. In the POA stage, WB results showed that TGF‐β1 was significantly upregulated and INHBA was slightly increased; TGF‐β1 and INHBA synergistically initiated the canonical TGF‐β pathway, which may represent a compensatory protective response of the body to early cartilage injury [27]. At this stage, the phosphorylation level of p38 MAPK was slightly elevated, which mainly induced chondrocyte stress adaptation rather than pathological damage. In the MOA stage, TGF‐β1 expression began to decline, while INHBA expression further increased, and the pathway activation mode shifted to the INHBA‐dominated noncanonical p38 MAPK pathway. On one hand, activin A encoded by INHBA can generate ROS via the ACVR2B–NOX4 axis; thus, activating the positive feedback loop of SMAD2/3‐p38 [28]; on the other hand, INHBA‐regulated CXCL12 can cross‐activate p38 by binding to the CXCR4 receptor [29]. These two mechanisms jointly promote the transition of chondrocytes to a catabolic phenotype, accompanied by a significant increase in p‐p38 MAPK levels and upregulated expression of matrix‐degrading enzymes such as MMP13. In the EOA stage, INHBA remained highly expressed, and p38 MAPK continued to be hyperactivated even as TGF‐β1 returned to a level not significantly different from that of the control group. This phenomenon indicates that sustained high expression of INHBA can promote AKT phosphorylation, inhibit the transcriptional activity of FoxO1, and thereby reduce the expression of anti‐inflammatory factors and matrix synthesis genes, forming a vicious cycle of “damage leading to further activation” [30] and ultimately resulting in full‐thickness cartilage loss and subchondral bone sclerosis [31]. Notably, the temporal expression pattern of INHBA in this study is highly consistent with the trajectory of the OA chondrocyte phenotypic transition. Single‐cell pseudotime analysis showed that the region of high INHBA expression completely overlaps with the transition path from stable chondrocytes (HomC) to catabolic chondrocytes (FC/preHTC), and its expression level is positively correlated with the OARSI score of cartilage lesions. This further confirms that INHBA is not only a molecular marker for OA progression but also a functional regulatory factor driving chondrocyte fate transition, and its dynamic expression changes can serve as a potential biological indicator for OA staging diagnosis.

Through drug repurposing, molecular docking, and molecular dynamics simulation, this study provides potential intervention strategies for INHBA‐mediated OA cartilage damage, and combined with the latest targeted research, highlights the feasibility and innovation of INHBA as a therapeutic target for OA. Regarding potential therapeutic compounds, progesterone showed a significant correlation with up to seven core genes including *INHBA*, suggesting that it may alleviate OA cartilage inflammation and fibrosis by inhibiting INHBA expression [32], which offers a new direction for hormonal regulation of the OA immune microenvironment. Molecular docking and 100 ns molecular dynamics simulation results for the clinically commonly used drug celecoxib showed that it not only exerts anti‐inflammatory effects through the classic COX‐2 inhibition pathway but also can stably bind to INHBA and directly inhibit the activin A activity of INHBA. This dual mechanism combining anti‐inflammatory effects and direct target inhibition explains why celecoxib has both pain‐relieving and cartilage damage‐delaying effects in clinical OA treatment [33] and provides a molecular‐level theoretical basis for optimizing the clinical application of existing OA drugs.

Although this study clarified the core regulatory role of the INHBA–TGF‐β/p38 MAPK axis in OA progression, it still has some limitations that should be acknowledged. First, the causal relationship between INHBA and OA progression requires further experimental validation. The current evidence is mainly based on correlation analysis and in silico functional perturbation. Definitive causal evidence requires additional gain‐ and loss‐of‐function experiments in chondrocytes and conditional knockout mouse models to directly demonstrate that INHBA drives the sequential activation of the TGF‐β/p38 MAPK pathway and subsequent cartilage damage. Second, the machine learning analysis was based on relatively small transcriptomic datasets, which increases the risk of overfitting. Although we adopted a NCV framework and validated the results in multiple independent cohorts, the model should be regarded as an exploratory tool for gene screening rather than a clinical predictive model. Larger multicenter cohorts with comprehensive clinical information are needed to construct a robust prediction model in the future. Third, further experimental validation using human specimens is required. Although we have provided extensive multicohort validation using human clinical transcriptomic data, the experimental validation of INHBA expression and function was only performed in rat OA models and in vitro chondrocyte models. Fourth, the scope of this study is limited. We did not investigate the paracrine regulatory role of INHBA in synovial tissue, and the validation of candidate therapeutic drugs only reached the molecular level. Future studies will address these issues using synovial–cartilage coculture models and in vivo intervention experiments.

## 5. Conclusion

In summary, through multidimensional experimental and literature evidence, this study indicates that INHBA is a key hub for immune microenvironment dysregulation and temporal pathway activation in OA, which may mediate the functional switch of the TGF‐β/p38 MAPK pathway from early compensation to late‐stage damage and ultimately contribute to cartilage degeneration in OA.

## Author Contributions

Jiahao Sun and Qi Sun designed this study. Jiahao Sun performed the bioinformatics analysis and analyzed the data. Yiqiang Chen and Qi Sun drafted the manuscript. Xu Cui and Xin Li made important contributions to data acquisition and integration. Jiahao Sun and Qi Sun completed the literature review. Wenpeng Xie and Yongkui Zhang reviewed and revised the manuscript.

## Funding

This study was supported by the National Natural Science Foundation of China (Grant 82575087).

## Disclosure

All authors contributed to this paper and approved the submitted version.

## Ethics Statement

The animal experiment was approved by the Animal Ethics Committee of the Affiliated Hospital of Shandong University of Traditional Chinese Medicine (Approval Number SDSZYYAWE20240408001, dated April 08, 2024). All procedures were performed in accordance with the institutional guidelines for the care and use of laboratory animals. For the bioinformatics analysis, publicly available and deidentified data from the GEO database were used, the use of which was approved, and the requirement for ethical approval was waived by the Animal Ethics Committee of the Affiliated Hospital of Shandong University of Traditional Chinese Medicine.

## Consent

Informed consent was not required for this study because it utilized deidentified, publicly available genomic data.

## Conflicts of Interest

The authors declare no conflicts of interest.

## Supporting Information

Additional supporting information can be found online in the Supporting Information section.

## Supporting information


**Supporting Information 1** Table 1: Basic information of all GEO datasets used in this study, including dataset grouping, GEO accession number, disease status, sample size, tissue source, and microarray platform.

**Supporting Information 2** Figure 1: Rationale for selecting the number of principal components (PCs) in single‐cell RNA sequencing analysis.

## Data Availability

The data that support the findings of this study are available from the corresponding author upon reasonable request.
