# Correction to “Immune Gene *INHBA* is Associated With Osteoarthritic Cartilage Damage and May Mediate the Temporal Activation of the TGF‐β/p38 MAPK Pathway: Integrating Multiomics Machine Learning and Experimental Validation”

**DOI:** 10.1155/mi/9857415

**Published:** 2026-08-26

J. Sun, Q. Sun, Y. Chen, et al., “Immune Gene *INHBA* is Associated With Osteoarthritic Cartilage Damage and May Mediate the Temporal Activation of the TGF‐β/p38 MAPK Pathway: Integrating Multiomics Machine Learning and Experimental Validation,” *Mediators of Inflammation* 2026 (2026): 8787726, https://doi.org/10.1155/mi/8787726.

In the above‐mentioned article, there was an error in the statement regarding co‐first authorship. The incorrect statement is as follows:

“Jiahao Sun and Qi Sun should be listed as co‐first authors.”

The correct statement should read as follows:

“Jiahao Sun, Qi Sun, and Yiqiang Chen contributed equally to this work and should be listed as co‐first authors.”

We apologize for this error.

