# Supplementary material for: Immune Gene INHBA is Associated With Osteoarthritic Cartilage Damage and May Mediate the Temporal Activation of the TGF‐β/p38 MAPK Pathway: Integrating Multiomics Machine Learning and Experimental Validation
Source: Mediators Inflamm. 2026 Jun 30;2026:8787726. doi: 10.1155/mi/8787726 (PMC13317150; doi:10.1155/mi/8787726)
Supplement: Supplementary file 1 — Supporting Information 1 Table 1: Basic information of all GEO datasets used in this study, including dataset grouping, GEO accession number, disease status, sample size, tissue source, and microarray platform. [file MI-2026-8787726-s001.docx]

Supplementary Table 1: GEO Data

| Group | GSE Series | Disease | Sample | Source | Platform |
| --- | --- | --- | --- | --- | --- |
| Data-train | GSE169077 | OA | 6 OA patients and 5 normal controls | Cartilage | GPL96 |
| Data-train | GSE178557 | OA | 4 OA patients and 4 normal controls | Cartilage | GPL13497 |
| Data-test | GSE235610 | OA | 6 OA patients and 6 normal controls | Cartilage | GPL24676 |
| Data-test | GSE246425 | OA | 8 OA patients and 4 normal controls | Cartilage | GPL24676 |
| Single-cell sequencing | GSE152805 | OA | 3 OA patients and 3 normal controls | Cartilage | GPL20301 |
