# Supplementary material for: Immune Gene INHBA is Associated With Osteoarthritic Cartilage Damage and May Mediate the Temporal Activation of the TGF‐β/p38 MAPK Pathway: Integrating Multiomics Machine Learning and Experimental Validation
Source: Mediators Inflamm. 2026 Jun 30;2026:8787726. doi: 10.1155/mi/8787726 (PMC13317150; doi:10.1155/mi/8787726)
Supplement: Supplementary file 2 — Supporting Information 2 Figure 1: Rationale for selecting the number of principal components (PCs) in single‐cell RNA sequencing analysis. [file MI-2026-8787726-s002.pdf]

A B

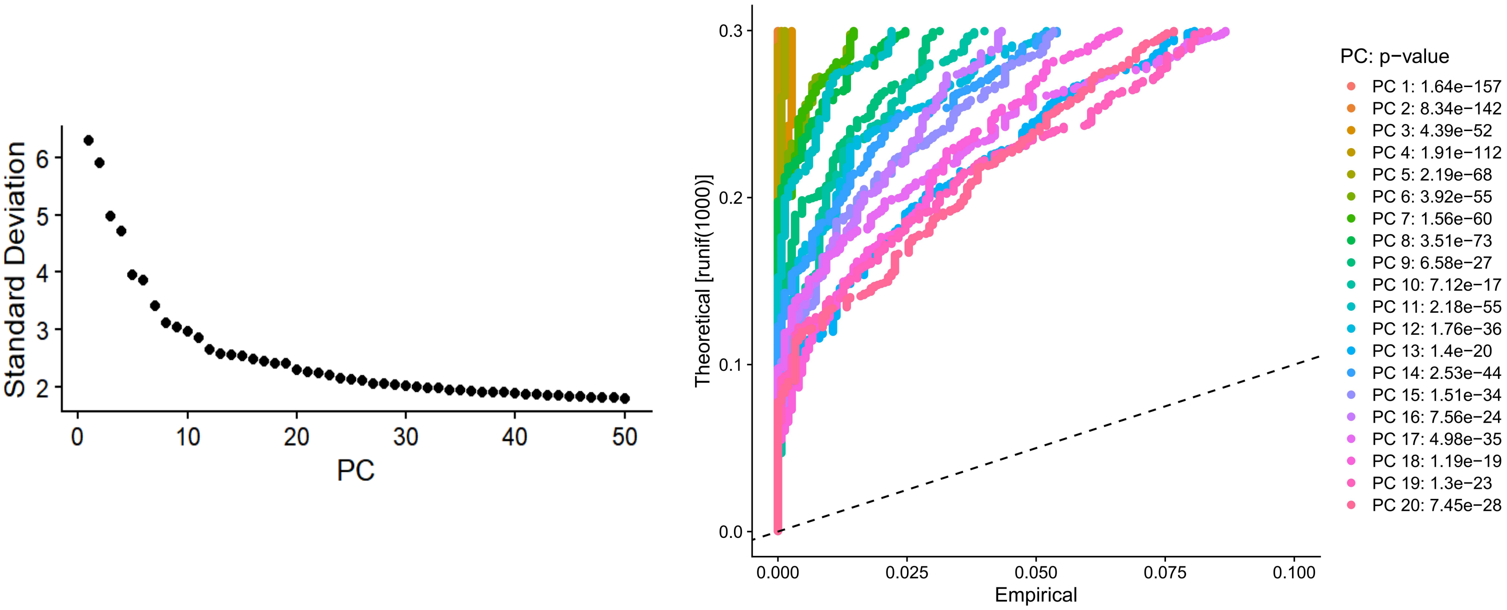

Figure S1 Rationale for selecting the number of principal components in single-cell PCA analysis.

A:JackStraw plot of PCA analysis, showing the statistical significance (p-values) of each principal component. The first 20 PCs are all at an extremely significant level (p <0.001), verifying the reliability of PCs in capturing biological variation;B:Elbow Plot of PCA analysis, showing the trend of principal component standard deviation with the number of principal components. The curve reaches a plateau after the 20th PC, jointly determining the optimal number of principal components as 20. Abbreviations: PC, principal component; PCA, principal component analysis.
